# *Acinetobacter baumannii* Lipopolysaccharide Influences Adipokine Expression in 3T3-L1 Adipocytes

**DOI:** 10.1155/2017/9039302

**Published:** 2017-07-05

**Authors:** Yuka Unno, Yoshinori Sato, Satoshi Nishida, Akiyo Nakano, Ryuichi Nakano, Tsuneyuki Ubagai, Yasuo Ono

**Affiliations:** ^1^Department of Microbiology and Immunology, Teikyo University School of Medicine, 2-11-1 Kaga, Itabashi, Tokyo 173-8605, Japan; ^2^Department of Microbiology and Infectious Diseases, Nara Medical University, 840 Shijo-cho, Kashihara, Nara 634-8521, Japan

## Abstract

*Acinetobacter baumannii* is one of the most important nosocomial opportunistic pathogen worldwide. In addition, obesity has been associated with an increased risk of nosocomial infection, suggesting that there may be an association between *A. baumannii* and white adipose tissue. However, the effects of *A. baumannii* on adipocytes have not been well studied at the molecular level. Here, we investigated the potential role of *A. baumannii*-derived lipopolysaccharides (LPS) as signaling molecules that affect adipocyte functionality. We tested the effect of increasing concentrations of *A. baumannii*-derived LPS (10, 100, or 1000 ng/mL) on the 3T3-L1 adipocyte cell line. Exposure to LPS was found to increase the expression of several adipokines (e.g., MIP-2, MCP-1, TNF-*α*, IL-6, lipocalin-2, and FABP4) in 3T3-L1 adipocytes and significantly reduced the expression of leptin and adiponectin. The effects of *A. baumannii*-derived LPS on MIP-2 expression were similar in comparison with that of LPS prepared from *Pseudomonas aeruginosa* and *Escherichia coli* in our cell culture-based system. This study suggests that *A. baumannii*-derived LPS functions as a signaling molecule that impacts the inflammatory function of white adipose tissue on the level of gene expression.

## 1. Introduction

*Acinetobacter baumannii* is a widespread gram-negative bacterium that primarily causes pulmonary, urinary tract, bloodstream, and surgical wound infections [1]. These bacteria easily and rapidly develop resistance to multiple antibiotics, and the presence of bacteremia in infected patients is associated with a poor prognosis [2]. Thus, *A. baumannii* is one of the most challenging pathogens for healthcare institutions worldwide [1–3]. Furthermore, *A. baumannii* is thought to possess only limited number of virulence factors and does not produce any known cytotoxins, suggesting that the pathogenetic mechanism of *A. baumannii* is poorly understood [4–6]. The opportunistic pathogen has lipopolysaccharides (LPS) in the outer membrane and may play an important role in the pathogenesis [4, 6].

Obesity is defined as excess adipose tissue. Adipose tissues function as key endocrine organs by releasing numerous proinflammatory and anti-inflammatory cytokines, as well as families of growth factors [7–9]. Thus, adipose tissue may represent an important site of inflammation. Recent studies have indicated that obesity is associated with an increased risk of nosocomial infections, such as a surgical wound, pulmonary, and urinary tract infections, and might contribute to an increase in future cases of sepsis [10, 11]. Furthermore, a more recent study focused on *A. baumannii* demonstrated that an altered nutritional status, resulting from hyperalimentation and obesity, could promote *A. baumannii* infection by reducing physiologic defense mechanisms [12]. These evidences suggest that *A. baumannii* and white adipose tissue may have a reciprocal relationship, and *A. baumannii* infection in obese patients may be associated with an increased risk of future sepsis.

The ability of LPS from several bacterial strains (i.e., *Escherichia coli*, *Neisseria meningitidis*, and *Salmonella enterica* serovar Minnesota) to induce systemic inflammation in response to a low-dose bacterial challenge has been well recognized [13–16]. In particular, *A. baumannii*-derived LPS (*A. b*-LPS) were reported to stimulate inflammatory signaling (e.g., interleukin-8, IL-8, and tumor necrosis factor, TNF-*α*) via human Toll-like receptors in human monocytic THP-1 cells [5]. These lines of evidence have raised the possibility that *A. baumannii* may have an influence on the physiological functionality of adipocytes, particularly in the synthesis and secretion of adipokines through LPS as a signaling molecule. Although we have previously demonstrated that *A. b*-derived LPS can influence the expression of several immunomodulatory genes in human neutrophils [17], the possible effects of *A. b*-LPS on adipocytes remain unknown.

Here, we examined the effects of *A. b*-derived LPS on adipokine expression in 3T3-L1 adipocytes in comparison with LPS isolated from other gram-negative bacteria, including pathogenic strains. Our results indicate that *A. b*-derived LPS affect adipocyte function, in particular, the upregulation of the chemoattractant and the inflammatory cytokines and the alteration of adipokine expression. These results support the hypothesis that an *A. baumannii* infection in obese patients may be associated with an increased risk of future sepsis.

## 2. Methods

### 2.1. Reagents

Mouse 3T3-L1 cells were obtained from the American Type Culture Collection (Manassas, VA, USA). Dulbecco's modified Eagle's medium (DMEM) with high glucose (11965-092), 1 mM sodium pyruvate, and 100 U/mL penicillin-100 *μ*g/mL streptomycin were from Thermo Fisher Scientific Inc. (Waltham, MA, USA). Fetal bovine serum (FBS) was obtained from Nissui Seiyaku (Tokyo, Japan). LPS from *E. coli* O111:B4 and LPS from *E. coli* O55:B5, 3-isobutyl-1-methylxanthine (IBMX), insulin, and dexamethasone were obtained from Sigma-Aldrich (St. Louis, MO). Mouse CXCL2/MIP-2 DuoSet ELISA was obtained from R&D Systems (Minneapolis, MN). All the reagents used were of the highest grade available from commercial sources.

### 2.2. Bacterial Strains

*A. baumannii* ATCC19606 and *P. aeruginosa* PAO1 were used as reference strains. The multidrug-resistant *P. aeruginosa* (MDRP) strain and multidrug-resistant *A. baumannii* (MDRA) strain were clinical isolates from the Teikyo University hospital. These bacteria were grown for 16 h at 37°C on Luria-Bertani (LB) agar (Wako Pure Chemical Industries). A single colony of the bacteria on the LB agar was used for the large-scale culture of the bacteria in LB broth (Wako Pure Chemical Industries). After incubating with shaking at 37°C, the bacteria in the midexponential phase was harvested by centrifugation, froze in liquid nitrogen, and stored at −20°C until use. In total, 50 L of the bacteria liquid culture was required for 5 g of the bacteria pellet. We contracted a company (Wako Pure Chemical Industries) to extract and purify LPS from these gram-negative bacteria using phenol and ultracentrifugation [17].

### 2.3. Cell Culture and Treatment

Unless otherwise specified, cell culture and further analyses were conducted at 37°C in an atmosphere comprising of 5% CO_2_ and 95% air. 3T3-L1 cells were maintained as subconfluent cultures in DMEM, supplemented with 25 mM glucose, 10% FBS, 1 mM sodium pyruvate, and 100 U/mL penicillin-100 *μ*g/mL streptomycin (medium A). These cells were seeded at a density of 4 × 10^5^ cells/well in 35 mm dishes and cultured for 3 days with 10 *μ*g/mL insulin, 0.25 *μ*g/mL dexamethasone, and 0.5 mM IBMX in medium A. The cells were further incubated for 5 days in medium A. The medium was changed every 2 days, as described previously [7]. The differentiated adipocytes thus prepared were characterized on day 8 by assessing the intracytoplasmic accumulation of lipid droplets; more than 80% of these adipocytes were stained positive with Oil red O [18]. These differentiated 3T3-L1 adipocytes were incubated for 0, 1, 24, 48, or 72 h in 35 mm dishes containing medium A with 100 ng/mL LPS. These cells were used for measuring adipokine expression levels, immunoblot analyses, and real-time quantitative PCR analyses, as described below.

### 2.4. RNA Preparation and Complementary DNA Synthesis

3T3-L1 cells were treated with LPS for 1 h at 37°C. As control, saline (the solvent for the LPS) adjusted to a concentration of 0.1% was used. Total RNA of 3T3-L1 cells was extracted using the RNeasy mini kit (Qiagen, Dusseldorf, Germany). An Agilent 2100 Bioanalyzer (Agilent Technologies, Waldbronn, Germany) was used to determine the quantity and quality of the total RNA samples. Total RNA was reverse-transcribed to cDNA following the manufacturer's instructions using the SuperScript® VILO™ cDNA Synthesis Kit (Life Technologies). Briefly, 2 *μ*g of total RNA was incubated with 4 *μ*L of the 5 × VILO reaction mix and 2 *μ*L of the SuperScript enzyme mix in a 20 *μ*L reaction volume at 25°C for 10 min, followed by further incubation at 42°C for 120 min. The reaction was terminated by heating to 85°C for 5 min. The cDNA product was diluted to a final volume of 40 *μ*L with deionized water and used for real-time quantitative PCR analysis.

### 2.5. Real-Time Quantitative PCR Analysis

Real-time quantitative PCR was performed to determine gene expression levels of target genes in mouse 3T3-L1 cells using ABI7300 real-time PCR System (Applied Biosystems; Foster City, CA). Real-time PCR reactions were performed in 10 *μ*L reaction volumes containing the following components: 1 *μ*L of the cDNA solution, 5 *μ*L of the SYBR® Green PCR Master Mix (Applied Biosystems; Foster City, CA), 300 nM of each primer (real-time PCR primer set for adipose (mouse); Hokkaido System Science Co., Ltd.), and deionized water. The sequence of the forward primer for macrophage inflammatory protein-2 (MIP-2) was 5′- GCTGTCCCTCAACGGAAGAA-3′, and that of the reverse primer was 5′- CAGGTACGATCCAGGCTTCC-3′. The sequence of the forward primer for lipocalin-2 was 5′-CCACCACGGACTACAACCAG-3′, and that of the reverse primer was 5′-CCTTCAGTTCAGGGGACAGC-3′. The sequence of the forward primer for TLR4 was 5′- CTCTGGGGAGGCACATCTTC-3′, and that of the reverse primer was 5′- TCAGGTCCAAGTTGCCGTTT-3′. The sequence of the forward primer for the endothelial nitric oxide synthase (eNOS) sequence was 5′- GGTTGCAAGGCTGCCAATTT-3′, and that of the reverse primer was 5′- TAACTACCACAGCCGGAGGA-3′. The cDNA amplification conditions were 95°C for 2 min, 40 cycles at 95°C for 15 sec, 60 sec at the annealing temperature 60°C. The target gene expression levels were normalized to the internal control (*β*-actin). Relative expression levels were calculated using the formula 2 − (*δδ*Ct), where *δδ*Ct is [Ct(target gene) − Ct(*β* − actin)]tested condition − [Ct(target gene) − Ct(*β* − actin)] control condition and Ct is the cycle at which the threshold is crossed. RT-PCR analysis was performed in triplicate on a mean of at least three separate experiments.

### 2.6. MIP-2 Measurement

After treating the adipocytes with LPS, the culture medium was collected and centrifuged at 10,000g for 10 min at 4°C, and the cells were solubilized with 1.0 mL of 0.1 N NaOH to determine the protein concentration using the bicinchoninic acid protein assay kit (Pierce, Rockford, IL). The amounts of MIP-2, the rodent equivalent of human IL-8, secreted in the supernatant were determined using a sandwich ELISA kit, with recombinant mouse MIP-2 as the standard, according to the manufacturer's instructions. Corrections to MIP-2 concentrations were made according to the protein content of the cells cultured in each well. The minimal detectable MIP-2 level in this assay was 15.6 pg/mL.

### 2.7. Statistical Analysis

The data was expressed as means ± SD. The program Prism 6 (GraphPad Software, Inc., San Diego, CA, USA) was used to perform the statistical analysis. Comparisons between groups were performed by one-way analysis of variance (ANOVA) and Dunnett posttest. Differences were considered significant when *P* < 0.05.

## 3. Results

### 3.1. Exposure to *A. b*-LPS Increases the Expression of Chemoattractant and Inflammatory Cytokines in 3T3-L1 Adipocytes

**Figure 1:**
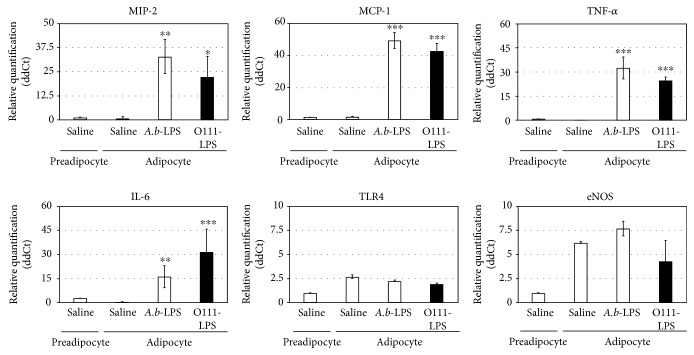
Effect of *A. baumannii*-LPS on adipokine expression including inflammatory cytokines and chemoattractants in 3T3-L1 adipocytes. Differentiated adipocytes were incubated for 1 h at 37°C with 100 ng/mL *A. baumannii*-LPS (ATCC 19606), 100 ng/mL *E. coli*-LPS (O111:B4), or saline and subjected to real-time PCR analyses to determine the mRNA expression levels of several adipokines, including MIP-2, TNF-*α*, IL-6, MCP-1, TLR4, and eNOS. Adipokine expression is presented as the ratio of viable cytokine expression in adipocytes treated with the different bacterial LPS versus the adipocytes treated with saline. All experiments were performed in triplicates, and the results are presented as the mean ± SD. ^∗^*P* < 0.05; ^∗∗^*P* < 0.01; ^∗∗∗^*P* < 0.005 (compared to adipocytes without LPS treatment).

To determine the effect of *A. b*-LPS on adipocyte functionality, on day 8, 3T3-L1 adipocytes were incubated for 1 h in the presence or absence of 100 ng/mL *A. b*-LPS. The expression levels of the chemoattractant (e.g., MIP-2 and monocyte chemotactic protein 1, MCP-1) and inflammatory cytokines (e.g., TNF-*α* and IL-6) increased at the mRNA level in adipocytes treated with *A. b*-LPS compared to the control cells without LPS treatment (Figure 1). Treatment of the adipocytes with 100 ng/mL *A. b*-LPS produced a 33-fold induction of MIP-2 and TNF-*α*, a 49-fold induction of MCP-1, and a 15-fold induction of IL-6. Treatment of the adipocytes with *E. coli* O111:B4 (O111-LPS) exhibited the same effect on inflammatory cytokine expression. Conversely, mRNA expression levels of TLR4 and eNOS were not affected by both *A. b*-LPS and O111-LPS.

### 3.2. Effect of *A. b*-LPS on Adipokine Expression in 3T3-L1 Adipocytes

**Figure 2:**
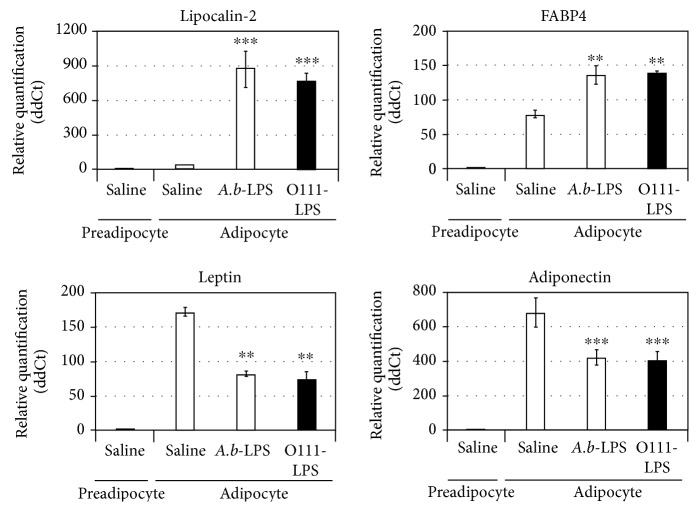
Effect of *A. baumannii*-LPS on adipokine expression in 3T3-L1 adipocytes. Differentiated adipocytes were incubated for 24 h with 100 ng/mL *A. baumannii*-LPS (ATCC 19606), 100 ng/mL *E. coli*-LPS (O111:B4), or saline and subjected to real-time PCR analysis to determine the mRNA levels of several adipokines, including lipocalin-2, FABP4, leptin, and adiponectin. Adipokine expression is presented as the ratio of viable adipokine expression in adipocytes treated with the LPS from each of the bacterial strains versus the adipocytes treated with saline. All experiments were performed in triplicates, and the results are shown as mean ± SD. ^∗∗^*P* < 0.01; ^∗∗∗^*P* < 0.005 (compared to adipocytes without LPS treatment).

The expression of a large number of cytokines, such as lipocalin-2, FABP4, leptin, and adiponectin, are markedly induced during the differentiation of preadipocytes into adipocytes [19, 20]. Clinical and experimental studies indicate an important role of lipocalin-2, FABP4, and leptin as inflammatory adipokines associated with obesity and related complications [20–22]. Unlike several other adipokines, adiponectin has been associated with insulin-sensitizing and anti-inflammatory properties [20, 23]. Therefore, we next evaluated the effect of *A. b*-LPS on the expression of these adipokines in 3T3-L1 adipocytes by incubating cells for 1 h and 24 h in the absence or presence of *A. b*-LPS. After 24 h treatment with *A. b*-LPS, a significant increase in lipocalin-2 and FABP4 was observed, whereas mRNA levels of leptin and adiponectin reduced in 3T3-L1 adipocytes (Figure 2). Furthermore, the expression of lipocalin-2, leptin, and adiponectin was not affected by *A. b*-LPS for at least 1 h (data not shown).

### 3.3. MIP-2 mRNA Was Upregulated by *A. b*-LPS in 3T3-L1 Adipocytes

**Figure 3:**
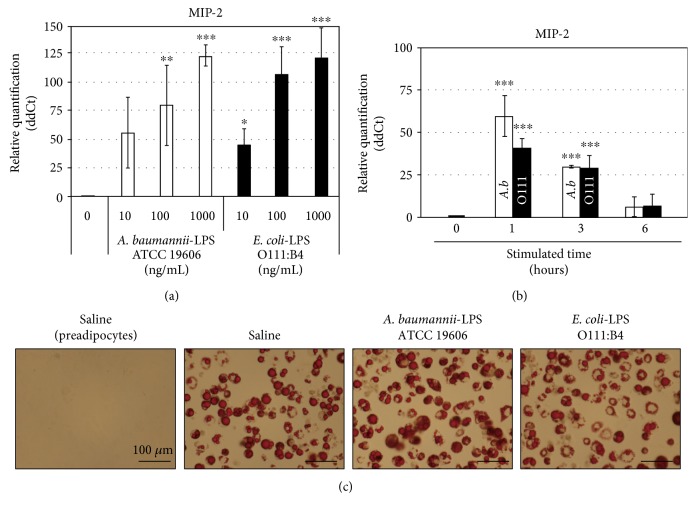
Induction of MIP-2 by *A. baumannii*-LPS in 3T3-L1 adipocytes. The dose-dependent effect of *A. baumannii*-LPS was examined via the MIP-2 expression in adipocytes. The adipocytes were incubated for 1 h in fresh medium containing 10, 100, or 1000 ng/mL of *A. baumannii*-LPS (ATCC 19606), (a) *E. coli*-LPS (O111:B4), or saline and were subjected to real-time PCR analyses for the detection of MIP-2 mRNA. (b) MIP-2 expression in adipocytes was measured at the indicated time points following treatment with 100 ng/mL *A. baumannii*-LPS (ATCC 19606) or 100 ng/mL *E. coli*-LPS (O111:B4). The expression of MIP-2 is presented as the ratio of viable MIP-2 expression in adipocytes treated with LPS versus those treated with saline at the indicated time points. All experiments were performed in triplicates, and the results are presented as the mean ± SD. (c) The microscopic images (×280 magnification) of 3T3-L1 adipocytes treated with LPS on day 8 via Oil-Red-O staining. 3T3-L1 adipocytes were differentiated in 35 mm dishes and subsequently stimulated with the LPS derived from several bacterial strains (1000 ng/mL) for 24 h. The morphological changes in adipocytes caused by LPS (1000 ng/mL) were assessed by optical microscopy. ^∗^*P* < 0.05; ^∗∗^*P* < 0.01; ^∗∗∗^*P* < 0.005 (compared to adipocytes without LPS treatment).

IL-8 is the human equivalent of rodent MIP-2 and a potential chemoattractant for neutrophil infiltration into adipose tissue secreted by adipocytes in models of obesity [24–26]. Therefore, we focused on the alteration of MIP-2 expression by *A. b*-derived LPS (*A. b*-LPS) and stimulated differentiated adipocytes with various concentrations of *A. b*-LPS (10, 100, or 1000 ng/mL) for 1 h. The level of MIP-2 mRNA in adipocytes was upregulated by *A. b*-LPS in a dose-dependent manner, with a maximum increase (125-fold) at 1000 ng/mL, compared to the levels exhibited by the untreated cells (Figure 3(a)). Furthermore, the time course of MIP-2 expression induced by *A. b*-LPS was compared with the LPS of O111-LPS. MIP-2 expression was maximum at the end of the 1 h time point and subsequently decreased. After 1 h, *A. b*-LPS induced a 60-fold upregulation of MIP-2 expression, but at 3 h and 6 h, the expression level had decreased to 30 and 6.3-fold, respectively (Figure 3(b)). MIP-2 expression in 3T3-L1 adipocytes treated with 1000 ng/mL O111-LPS was upregulated by 125-fold, similar to that exhibited by *A. b*-LPS (Figure 3(a)). Under these conditions, the LPS used for stimulation had no effect on cytotoxicity, as assessed by both Oil-Red-O staining (Figure 3(c)) and a WST-8 cell proliferation assay (data not shown).

### 3.4. Effect of *A. baumannii*-LPS on MIP-2 Secretion from 3T3-L1 Adipocytes

**Figure 4:**
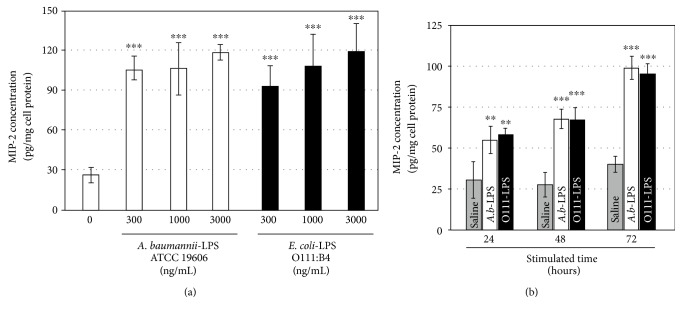
Effect of *A. baumannii*-LPS on MIP-2 secretion from 3T3-L1 adipocytes. (a) The dose-dependent effect of *A. baumannii*-LPS on MIP-2 secretion from adipocytes. The 3T3-L1 cells were seeded at a density of 4 × 10^5^ cells/well in 35 mm dishes, and the cells were differentiated into adipocytes in MIX-Diff medium. The adipocytes were then incubated for 72 h in fresh medium containing 300, 1000, and 3000 ng/mL of *A. baumannii*-LPS (ATCC 19606) or *E. coli*-LPS (O111:B4) and measured for the level of MIP-2 protein in the culture medium by ELISA. (b) MIP-2 secretion from adipocytes was measured at the indicated time points following treatment with 1000 ng/mL *A. baumannii*-LPS (ATCC 19606), 1000 ng/mL *E. coli*-LPS (O111:B4), or saline. All experiments were performed in triplicates, and the results are shown as the mean ± SD. ^∗∗^*P* < 0.01 (compared to cells without LPS treatment). ^∗∗^*P* < 0.01; ^∗∗∗^*P* < 0.005 (compared to adipocytes without LPS treatment).

We next explored whether MIP-2 secretion increased in 3T3-L1 adipocytes stimulated with *A. b*-LPS. The differentiated adipocytes were incubated for 72 h with or without *A. b*-LPS (300, 1000, or 3000 ng/mL). A small amount of MIP-2 was secreted into the culture medium, with the basal level of 27.2 pg/mg of cellular MIP-2 protein. Moreover, MIP-2 secretion from 3T3-L1 adipocytes treated with 3000 ng/mL *A. b*-LPS increased by 4.4-fold. Furthermore, incubation with 3000 ng/mL O111-LPS also increased MIP-2 expression by 4.4-fold (Figure 4(a)). Next, the time course of MIP-2 secretion by *A. b*-LPS was compared with that of the untreated cells. At 24 h, MIP-2 secretion exhibited a 1.8-fold increase following treatment with 1000 ng/mL *A. b*-LPS, and at both 48 h and 72 h, MIP-2 secretion was upregulated by 2.5-fold (Figure 4(b)). Together, these results indicate that *A. b*-LPS and O111-LPS can enhance MIP-2 expression at the mRNA level, leading to MIP-2 secretion from the stimulated adipocytes.

### 3.5. Adipokine Expression following Stimulation with LPS Isolated from Nosocomial Opportunistic Pathogens

**Figure 5:**
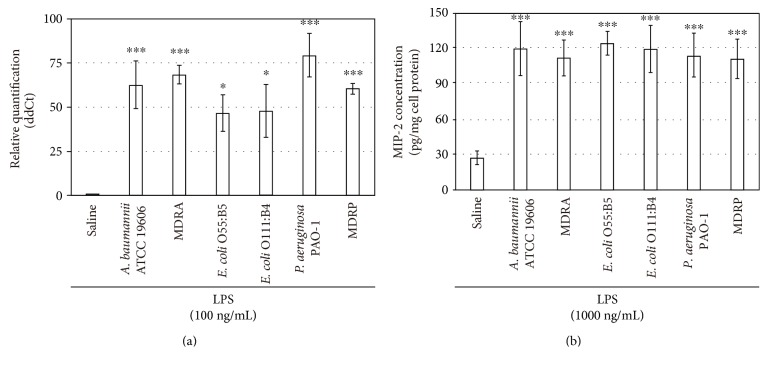
Effect of LPS prepared from six strains of gram-negative bacteria on MIP-2 expression in 3T3-L1 adipocytes. (a) Differentiated adipocytes were incubated for 1 h with 100 ng/mL LPS prepared from six strains of gram-negative bacteria, including *A. baumannii* ATCC 19606, MDRA, *E. coli* O55:B5, *E. coli* O111:B4, *P. aeruginosa* PAO1, and MDRP. The cells were then subjected to a real-time PCR analysis for MIP-2 expression. (b) Differentiated adipocytes were incubated for 48 h in complete fresh medium containing 1000 ng/mL LPS prepared from six strains of gram-negative bacteria or saline, and the MIP-2 levels in the culture medium were measured by an ELISA. All experiments were performed in triplicates, and the results are presented as the mean ± SD. ^∗^*P* < 0.05; ^∗∗∗^*P* < 0.005 (compared to adipocytes without LPS treatment).

Finally, we tested the effect of six types of LPS isolated from *A. baumannii,* MDRA, *E. coli* O55:B5, *E. coli* O111:B4, *P. aeruginosa* PAO1, and MDRP on MIP-2 expression in 3T3-L1 adipocytes (Figures 5(a) and 5(b)). At the mRNA level, *A. b*-LPS treatment for 1 h resulted in a 62-fold upregulation of MIP-2 expression compared with the control cells that did not receive LPS treatment. Stimulation with LPS derived from the other bacteria also significantly increased MIP-2 expression (68-fold for MDRA, 47-fold for *E. coli* O55:B5, 48-fold for *E. coli* O111:B4, 77-fold for PAO1, and 60-fold for MDRP; Figure 5(a)). ELISA studies revealed that the LPS prepared from these bacterial strains also increased the level of MIP-2 secretion from 3T3-L1 adipocytes by 4.4-fold for *A. baumannii*, 4.1-fold for MDRA, 4.6-fold for *E. coli* O55:B5, 4.4-fold for *E. coli* O111:B4, 4.2-fold for PAO1, and 4.1-fold for MDRP under the LPS treatment for 48 h (Figure 5(b)). A comparison of the induced MIP-2 expression between the six LPSs revealed no significant differences. Under these conditions, the LPS prepared from these bacteria exhibited no effect on cytotoxicity, as assessed by the WST-8 assay described above (data not shown).

## 4. Discussion

In the present study, we found that *A. b*-LPS mediates the upregulation of chemoattractants and inflammatory cytokines, including MIP-2, MCP-1, TNF-*α*, and IL-6, at the mRNA level in 3T3-L1 adipocytes. Furthermore, *A. b*-LPS reduce the expression of leptin and adiponectin in 3T3-L1 adipocytes. A previous report indicated that an altered nutritional status due to obesity could promote an *A. baumannii* infection by reducing physiologic defense mechanisms [12]. Thus, taken together with our results, these findings imply that *A. b*-LPS may act as a direct signaling molecule that impacts the inflammatory function of adipose tissue.

Adipose tissue is an important source of TNF-*α* and other cytokines [19]. In adipose tissue, the adipocytes are surrounded by immune cells; every gram of adipose tissue contains 1-2 million adipocytes and 4–6 million stromal-vascular cells, of which more than half are leukocytes [27]. In addition, a number of studies demonstrate that neutrophil infiltration into adipose tissue precedes macrophage infiltration during a classical immune response [28, 29]. In this study, we demonstrated that *A. b*-LPS induce the upregulation of MIP-2, a strong chemoattractant of neutrophils, and MCP-1 in 3T3-L1 adipocytes (Figures 1 and 3). Furthermore, we have previously demonstrated that *A. b*-LPS can influence the expression of several proinflammatory and immunomodulatory genes in human neutrophils [17]. Thus, we speculate that when the neutrophils and macrophages infiltrate into obese adipose tissue induced by the chemoattractant, the cells are also an important source of inflammatory cytokines. These inflammatory cytokines from adipose tissue may evoke systemic inflammation and a cytokine storm.

There is an in vitro study demonstrating a significant decrease in leptin mRNA in 3T3-L1 adipocytes stimulated by LPS [30], as similar to our results (Figure 2). On the other hand, there are in vivo studies demonstrating that the plasma level of leptin was elevated after the intraperitoneal injection of LPS into mice [31, 32]; these data seem to be inconsistent with the present interpretation in our cell-based system. In a different context, there are in vivo and in vitro evidences for the involvement of TNF-*α* in the induction of leptin by LPS [31]. A systemic inflammatory response induced by LPS may predominate over the LPS-dependent leptin regulation possibly occurring in the local area of inflammatory responses. Further studies are necessary to understand the complex relationship between the local cellular responses and the systemic responses of leptin to LPS.

*A. baumannii* has the propensity to accumulate mechanisms of antimicrobial resistance, leading to the rapid acquisition of pan-drug resistance. Because it is difficult to eradicate multidrug-resistant bacteria (e.g., MDRA and MDRP) at the site of infection, persistent infections caused by these gram-negative bacteria may develop following the chronic release of LPS. In our study, the LPS derived from MDRA and MDRP increased the expression of MIP-2 in 3T3-L1 adipocytes. Thus, our findings imply that the LPS derived from multidrug-resistant bacteria promote adipocyte inflammation.

Korneev et al. investigated that the structural variations in lipid A as an LPS structural component affect the ability of LPS from different bacteria to activate the innate immune response through TLR4 [33]. The ability of LPS isolated from *A. baumannii* 1053 (a clinical isolate from a patient with cystic fibrosis), to induce cytokine production (e.g., IL-6 and TNF), is similar to that of LPS isolated from *E. coli* O130 in murine bone marrow-derived macrophages [33]. In contrast, they demonstrated that the biological activity of LPS from the *P. aeruginosa* 2192 (a clinical isolate from a cystic fibrosis patient) did not induce measurable cytokine production [33]. The reasons for the difference in the effect of LPS are as follows: the impact of LPS steadily decreases with the number and the length of the acyl chains of lipid A. We previously demonstrated that gene expression levels in human neutrophils in response to LPS from drug-sensitive strains (*A. baumannii* or *P. aeruginosa* PAO-1) differ from that of LPS from their respective drug-resistant strains (MDRA and MDRP) [17]. Thus, we speculate that it is possible that these LPS subtypes contain different biochemical modifications and are structurally distinct. However, in the present study, the effects of *A. b*-LPS on the expression of MIP-2 were similar compared to those of LPS prepared from MDRA, MDRP, PAO1, and *E. coli* in our cell culture-based system (Figure 5); these results do not explain fully the development of antibiotic resistance or poor prognosis associated with *A. baumannii* infections as compared to other gram-negative bacteria. Further studies will be necessary to understand how even the smallest structural differences between very similar bacterial ligands may affect the activation of the inflammatory response in adipocytes. These studies may provide a mechanism to fine tune the inflammatory response of adipocytes and novel insight into the progression of obesity-associated systemic inflammation.

To our knowledge, this study is the first to investigate the biological activity of LPS derived from *A. baumannii* in adipocytes. *A. b*-LPS induces the upregulation of chemoattractants and inflammatory cytokines, including MIP-2, MCP-1, TNF-*α*, and IL-6, and also reduces the expression of leptin and adiponectin in 3T3-L1 adipocytes. These findings suggest that the LPS derived from *A. baumannii* may act as a direct signaling molecule that impacts the inflammatory function of adipose tissue and is associated with an increased risk of future septic events.
